# Microbial Species that Initially Colonize the Human Gut at Birth or in Early Childhood Can Stay in Human Body for Lifetime

**DOI:** 10.1007/s00248-020-01636-0

**Published:** 2021-01-07

**Authors:** Weizhong Li, Karen E. Nelson

**Affiliations:** 1 J. Craig Venter Institute, La Jolla, CA 92037 USA; 2 J. Craig Venter Institute, Rockville, MD 28050 USA

**Keywords:** Microbiome, Gut microbiome, Metagenomic strain variants, Microbiome persistence, Twin pairs

## Abstract

In recent years, many studies have described the composition and function of the human microbiome at different body sites and suggested a role for the microbiome in various diseases and health conditions. Some studies, using longitudinal samples, have also suggested how the microbiome changes over time due to disease, diet, development, travel, and other environmental factors. However, to date, no study has demonstrated whether the microorganisms established at birth or in early childhood, either transmitted from parents or obtained from the environment, can stay in the human body until adult or senior age. To directly answer this question is difficult, because microbiome samples at childhood and at later adulthood for the same individual will need to be compared and the field is not old enough to have allowed for that type of sample collection. Here, using a metagenomic approach, we analyzed 1004 gut microbiome samples from senior adults (65 ± 7.8 years) from the TwinsUK cohort. Our data indicate that many species in the human gut acquired in early childhood can stay for a lifetime until senior ages. We identified the rare genomic variants (single nucleotide variation and indels) for 27 prevalent species with enough sequencing coverage for confident genomic variant identification. We found that for some species, twin pairs, including both monozygotic (MZ) and dizygotic (DZ) twins, share significantly more rare variants than unrelated subject pairs. But no significant difference is found between MZ and DZ twin pairs. These observations strongly suggest that these species acquired in early childhood remained in these persons until senior adulthood.

**Supplementary Information:**

The online version contains supplementary material available at 10.1007/s00248-020-01636-0.

## Introduction

The human microbiome refers to the millions of microbial organisms that live on different body sites [1, 2]. These microbes are essential for maintaining human health by producing nutrients and vitamins, regulating the immune system, providing the host with beneficial molecules, protecting the gut barrier, and fighting off disease-causing pathogens. For healthy humans, the microbiome gets populated immediately at birth. After birth, various microbial species from mothers and from the surrounding environments quickly colonize the infants, especially in the gastrointestinal tract. This initial colonization is influenced by birth modes (vaginal delivery or C-section), use of antibiotics, breastfeeding, and several other factors [3–7]. The maternal gut has been found to be the largest source of colonizing bacteria in the gastrointestinal tract of healthy infants [8].

The composition of the human microbiome is thought to evolve and change as humans age. After birth, the infant’s gut microbiome undergoes a development phase for about a year, then a transition phase for another year, and then reaches a stable phase [9]. Youth, adult, and senior populations each harbor age-specific features in their microbiomes [10]. Gut microbiome composition can be disrupted due to acute diseases or infections [11], diet [12, 13], use of antibiotics [14] and other drugs [15], travel [16], and many other factors. However, the composition of an established gut microbiome has a tendency to restore after disruption [17]. The stability of the human gut microbiome has been shown by longitudinal studies. One study [18], which followed subjects for years, showed that some microbial species can stay in the human gut for many years. However, so far, no study has demonstrated whether the microorganisms established at birth or in early childhood, either transmitted from parents or obtained from the environment, remain in the human body until adulthood or senior age. To directly answer such a question is difficult, because microbiome samples at childhood and at later adulthood for the same individual would need to be collected and compared.

The microbial species in the human microbiome exhibit large genomic variations among different individuals. A large number of single nucleotide variations (SNVs), short insertions/deletions (indels), and structural variants from the same species have been found among different samples [19]. These metagenomic variations are very unique and can be used as signatures to identify individuals [20]. Here, we analyzed 1004 gut microbiome samples from senior adults (65 ± 7.8 years) from the TwinsUK cohort [21]. In this study, by analyzing the rare SNVs and indels in this metagenomic dataset, we can indirectly show whether species in the human gut acquired at birth or in early childhood can persist for a lifetime until senior ages.

The microbiome datasets from the TwinsUK cohort generated by either 16S rDNA sequencing or shotgun metagenomic sequencing have been analyzed in several different studies, which have suggested a heritability of the human microbiome and how genetic and environmental factors impact the gut microbiome, as well as the associations between the gut microbiome and metabolites in stool and blood samples [22–25]. In these studies, as well as other microbiome studies involving samples from twins [26], it has been repeatedly observed that the similarity of the microbiome between twin pairs is significantly higher than in unrelated subjects. These observations were explained by the host genetics, which also explains why the microbiome similarity within the monozygotic (MZ) twin pairs is greater than the dizygotic (DZ) twin pairs [22–24]. However, the previous studies have not really answered the question as to when these species shared by twin pairs started to colonize the gut.

In this study, we used the same dataset from the TwinsUK cohort, but focused on a set of rare SNVs and indels in the microbial species, and generated results that support the observation that the shared rare genomic variants between twin pairs are from species that colonize them at an early age.

## Results and Discussion

In this study, we re-analyzed the stool samples from 1004 individuals from the TwinsUK cohort that were sequenced using the shotgun metagenomic approach from our previous study [25]. This dataset includes 161 MZ twin pairs, 201 DZ twin pairs, and 280 individuals without a matching twin. We first identified a subset of abundant and common species for the analysis of SNVs and indels. We found 27 species that meet the following criteria: (1) present in more than 100 individuals, (2) present in at least 10 twin pairs, (3) average depth of sequencing coverage ≥ 2, (4) the fraction of genome covered by mapped metagenomic reads is ≥ 75%. These criteria ensure enough sample size for statistical analysis and sequencing coverage for reliable variant calls. The list of the 27 species and number of samples and number of twin pairs for these species are presented in Table S1.

SNVs and indels for these species were identified using the Varscan 2 program (see Materials and Methods). For each species, rare variants (SNVs or indels) that were only found in less than 20% of samples were kept for further analysis. On average, 171,316 ± 90,761 rare SNVs and 3308 ± 1424 rare indels were found per species (Table S1). For each species, a variant cutoff is defined so that among all the pairs of unrelated individuals having this species, only 1% of these pairs share more rare variants than this cutoff (Table S1). For example, the cutoff for *Methanobrevibacter smithii* is 971. It means that there is a 1% probability that a pair of unrelated individuals with *M. smithii* share ≥ 971 rare variants. Here, for a species, if two individuals meet this cutoff, we consider they share the same strain-specific variant signature (at a significance level of 0.01). Having strain-specific variant signatures indicates that the strains from the two individuals can be traced back to the same origin.

For some species, the frequency for the twin pairs sharing strain-specific variant signatures is much higher than 1%, which is the expected frequency for unrelated pairs. Exact binomial test (binom.test in R package) was used to calculate the *p* value for the observed number of twin pairs sharing strain-specific variant signature (Table 1). These species, in order of increasing *p* value, are *M. smithii*, *Bacteroides caccae*, *Bifidobacterium adolescentis*, *Alistipes putredinis*, *Bacteroides fragilis*, *Bacteroides dorei*, *Eubacterium hallii*, *Phascolarctobacterium faecium*, *Bacteroides uniformis*, *Blautia obeum*, *Bifidobacterium longum*, *Dialister invisus*, and *Akkermansia muciniphila.*

**Table 1:** Fraction of twin pairs that share strain-specific variants

Species	Number of twin pairs	Number of twin pairs sharing strain-specific variant signature ^a^	Frequency ^a^	*p* value
*Methanobrevibacter smithii*	22	8	0.3636	2.8E−11
*Bacteroides caccae*	42	6	0.1429	3.9E−06
*Bifidobacterium adolescentis*	25	5	0.2000	4.5E−06
*Alistipes putredinis*	114	7	0.0614	1.6E−04
*Bacteroides fragilis*	13	3	0.2308	2.7E−04
*Bacteroides dorei*	66	5	0.0758	5.4E−04
*Eubacterium hallii*	44	4	0.0909	9.9E−04
*Phascolarctobacterium faecium*	20	3	0.1500	1.0E−03
*Bacteroides uniformis*	201	8	0.0398	1.0E−03
*Blautia obeum*	71	4	0.0563	5.7E−03
*Bifidobacterium longum*	45	3	0.0667	0.01
*Dialister invisus*	54	3	0.0556	0.02
*Akkermansia muciniphila*	84	3	0.0357	0.05
*Escherichia coli*	13	1	0.0769	0.12
*Gemmiger formicilis*	16	1	0.0625	0.15
*Eubacterium siraeum*	22	1	0.0455	0.20
*Bacteroides vulgatus*	87	2	0.0230	0.22
*Subdoligranulum* sp. *APC924/74*	100	2	0.0200	0.26
*Eubacterium rectale*	50	1	0.0200	0.39
*Dorea longicatena*	54	1	0.0185	0.42
*Parabacteroides merdae*	32	0	0.0000	1.00
*Bacteroides* sp. *4_3_47FAA*	32	0	0.0000	1.00
*Ruminococcaceae bacterium TF06-43*	21	0	0.0000	1.00
*Coprococcus comes*	36	0	0.0000	1.00
*Barnesiella intestinihominis*	16	0	0.0000	1.00
*Streptococcus thermophilus*	13	0	0.0000	1.00
*Alistipes* sp. *AM16-43*	13	0	0.0000	1.00

^a^Frequency for the twin pairs sharing strain-specific variant signature

While it is expected that only 1% of pairs of unrelated individuals may share strain-specific variant signatures by chance, for *M. smithii*, 36% of twin pairs did. This suggests that for twin pairs where both individuals have *M. smithii*, 36% of them can be traced back to the same origin. Other species with high frequency for the twin pairs sharing strain-specific variant signatures include *B. fragilis* (23%), *B. caccae* (14%), and *E. hallii* (9%) (Table 1). This highly suggests that these particular strains shared by the twins originated from the same source and colonized the twins at their early ages. Otherwise, although the twins may preferably select similar microbial species in later adulthood while they are physically and geographically separated, it is difficult to explain why these strains share so many rare genomic variants. In addition, we did not find significant differences between MZ and DZ twin pairs.

Our observation about the strain-specific variant signatures shared between twins is highly connected with the heritability of the microbiome. Species with most genomic variants preserved in twins, such as *M. smithii*, are also among the list of heritable microorganisms shown in previous publications [18, 22–24]. In fact, if a species that is originally colonized both twins during childhood survived until their adulthood, this species is likely to be a heritable species. However, there are differences between heritability analysis and our analysis. Heritability was calculated based on the similarity of microbial species abundance between individuals’ microbiome [18, 22–24]. But in this study, species abundance was not considered, only the variants were compared. In heritability analysis, the effect of non-genetic factors (e.g., environment) on the data was modeled and removed [22–24], so the estimated heritability is only due to the genetic factors. Therefore, the heritable species were different between twins and unrelated pairs and were also different between MZ and DZ twins. In our analysis, we only observed the difference between twins and unrelated pairs, not between MZ and DZ twins. So, shared environment played an important role in addition to genetic factors in our analysis.

Some species, such as *Bacteroides vulgatus*, *Subdoligranulum* sp. *APC924/74*, and *Dorea longicatena*, showed no significant difference between twin pairs and unrelated pairs (Table 1), which indicates that these strains colonized in later adult stages when the twins were separated, or the strains had diverged too much to be detected by our genomic variant analysis, or had been replaced by related strains.

It is expected that microbes, after the initial colonization in the human gut, continue to evolve and diverge. However, to answer the question about how fast different species in the human microbiome evolve over lifetime, longitudinal samples are needed. So, we cannot address this question here. A previous study [18] with longitudinal samples for up to 5 years showed microbiome community divergence over time based on 16S rDNA sequencing data. In this study, we tried to see if there is a correlation between number of shared variants in twins and their age. However, we did not find any significant correlation. This suggested that although the species continued to evolve, the accumulation of genomic variants did not happen at a linear rate. Otherwise, we should have observed that older twins have less shared variants than younger twins.

In this study, using strain-level genomic variant analysis on one of the largest available human metagenomic datasets from the TwinsUK cohort, we estimate that some species in the human gut microbiome may stay for life after their initial colonization since early childhood. After many decades, the strain-specific genomic variant signatures can still be detected. To further understand how the species evolve and how their genomic variants occur over a lifetime, more investigations with longitudinal samples are needed.

## Materials and Methods

### Study Cohort and Samples

The subjects are from the TwinsUK adult twin registry, which includes about 14,000 subjects, predominantly females [21]. The stool samples were collected from a subset of subjects. Details are described in our previous study [25]. Briefly, stool samples from 1004 individuals (39 males, 965 females) of European ancestry were collected. These subjects (65.0 ± 7.8 years) were all living in the UK at the time of specimen collection. This dataset includes 161 monozygotic (MZ) twin pairs, 201 dizygotic (DZ) twin pairs, and 280 singletons. Data on the TwinsUK participants are available for research under managed access due to governance and ethical constraints and can be requested from http://twinsuk.ac.uk/resources-for-researchers.

### Metagenomic Sequencing

DNA extraction from stool samples, DNA library preparation, and metagenomic sequencing of the 1004 samples were also described in our previous study [25]. Sequencing of the stool samples yielded an average number of reads of 54 M per sample. The raw metagenomic sequences are available from the European Nucleotide Archive website (study accession number: PRJEB32731).

### Metagenomic Sequence Analysis

Raw reads were processed using Trimmomatic (version 0.36) [27] to trim low-quality bases (parameters: SLIDINGWINDOW:4:20 LEADING:3 TRAILING:3 MINLEN:80 MAXINFO:80:0.5). Only paired-end (PE) reads both of ≥ 80 bp were kept for analysis. High-quality PE reads were mapped to the human reference genome (hg38) with BWA-MEM (version 0.7.12) [28] with default parameters and removed, if they mapped concordantly with an alignment score of ≥ 60. We maintain a comprehensive microbiome reference genome database for mapping the metagenomic reads. This database was compiled and curated from NCBI Refseq genomes covering complete and draft bacteria, archaea, viruses, fungi, and microbial eukaryotes species. Currently, the database contains 27,115 representative genomes. Taxonomy profiles were determined through reference genome mapping using Centrifuge (version 1.0.4) [29] with default parameters.

After initial mapping using Centrifuge, we first filtered out mapped genomes from contamination. The depth of coverage (read length x number of mapped reads/genome length) and fraction of coverage (number of bases covered by mapped reads/genome length) for each mapped genome was calculated using the alignment file provided by Centrifuge. Some genomes were filtered out using the following criteria. In sequencing, the observed number of times a base is sequenced follows a Poisson distribution [30]. Given the observed depth of coverage calculated from the alignment file, the expected fraction of coverage is (1–1/(*e*^depth of coverage^)) [30]. If the observed fraction of coverage was smaller than 1/10 of the expected value, then it suggested aligned reads were piled up at a small fraction on the genome rather than uniformly distributed along the genome, the genome was removed.

Then relative taxonomy abundance was calculated based on the filtered mapping results.

### Genomic Variant Analysis

Our approach for genomic variants is similar to several existing methods that can analyze metagenomic data at strain-level such as StrainPhlAn [31], StrainEST [32], and MIDAS [33]. StrainPhlAn detects single nucleotide variation (SNV) based by mapping the reads to a set of conserved and unique species marker genes. StrainEST and MIDAS perform full-length genome SNV calling. Here, we implemented a novel approach for genomic variant analysis, including analysis of both SNVs and also indels.

First, we selected the species with at least 2× depth of coverage, based on the mapping data from the previous Centrifuge run. We then performed a second round of mapping to align the reads to these selected species using BWA-MEM [28] (parameters: *-*T 80). In this round of reads mapping, only one representative genome was used for one species. After mapping, we analyzed the BAM file using SAMtools (version 1.9) depth command [34] and calculated the depth of coverage and fraction of coverage for each genome. Genomes were removed if the depth of coverage is < 2 or fraction of coverage is < 0.75. SAMtools mpileup command was used to convert the BAM file to a mpileup file. Then, Varscan (version 2.4.2) pileup2snp [35] was used to call SNVs (parameters: --min-coverage 2 --min-reads2 2 --min-var-freq 0.66). Varscan pileup2indel was used to detect indels (parameters: --min-coverage 2 --min-reads2 2 --min-var-freq 0.66).

For one species, the detected SNVs and indels were collected for the samples that had this species at ≥ 2 depth of coverage and ≥ 0.75 fraction of coverage. And then from these collected variants, only rare SNVs and indels that exist in less than 20% of the samples were kept for further analysis, because common SNVs or indels are not discriminative in tracking strains between subjects.

## Electronic Supplementary Material

ESM 1 (DOCX 22 kb)

## Data Availability

The raw metagenomic sequences are available from the European Nucleotide Archive website (study accession number: PRJEB32731).

## References

[1] Gill SR, Pop M, DeBoy RT, Eckburg PB, Turnbaugh PJ, Samuel BS, Gordon JI, Relman DA, Fraser-Liggett CM, Nelson KE (2006). Metagenomic analysis of the human distal gut microbiome. Science.

[2] Human Microbiome Project Consortium (2012). Structure, function and diversity of the healthy human microbiome. Nature.

[3] Bokulich NA (2016). Antibiotics, birth mode, and diet shape microbiome maturation during early life. Sci. Transl. Med..

[4] Bäckhed F, Roswall J, Peng Y, Feng Q, Jia H, Kovatcheva-Datchary P, Li Y, Xia Y, Xie H, Zhong H, Khan MT, Zhang J, Li J, Xiao L, al-Aama J, Zhang D, Lee YS, Kotowska D, Colding C, Tremaroli V, Yin Y, Bergman S, Xu X, Madsen L, Kristiansen K, Dahlgren J, Wang J (2015). Dynamics and stabilization of the human gut microbiome during the first year of life. Cell Host Microbe.

[5] Li W, Tapiainen T, Brinkac L, Lorenzi HA, Moncera K, Tejesvi MV, Salo J, Nelson KE (2020) Vertical transmission of gut microbiome and antimicrobial resistance genes in infants exposed to antibiotics at birth. J. Infect. Dis. 10.1093/infdis/jiaa155 10.1093/infdis/jiaa155 PMC8514186 32239170

[6] Tapiainen T, Koivusaari P, Brinkac L, Lorenzi HA, Salo J, Renko M, Pruikkonen H, Pokka T, Li W, Nelson K, Pirttilä AM, Tejesvi MV (2019). Impact of intrapartum and postnatal antibiotics on the gut microbiome and emergence of antimicrobial resistance in infants. Sci. Rep..

[7] Ho NT, Li F, Lee-Sarwar KA, Tun HM, Brown BP, Pannaraj PS, Bender JM, Azad MB, Thompson AL, Weiss ST, Azcarate-Peril MA, Litonjua AA, Kozyrskyj AL, Jaspan HB, Aldrovandi GM, Kuhn L (2018). Meta-analysis of effects of exclusive breastfeeding on infant gut microbiota across populations. Nat. Commun..

[8] Ferretti P (2018). Mother-to-infant microbial transmission from different body sites shapes the developing infant gut microbiome. Cell Host Microbe.

[9] Stewart CJ, Ajami NJ, O’Brien JL, Hutchinson DS, Smith DP, Wong MC, Ross MC, Lloyd RE, Doddapaneni HV, Metcalf GA, Muzny D, Gibbs RA, Vatanen T, Huttenhower C, Xavier RJ, Rewers M, Hagopian W, Toppari J, Ziegler AG, She JX, Akolkar B, Lernmark A, Hyoty H, Vehik K, Krischer JP, Petrosino JF (2018). Temporal development of the gut microbiome in early childhood from the TEDDY study. Nature.

[10] Yatsunenko T, Rey FE, Manary MJ, Trehan I, Dominguez-Bello MG, Contreras M, Magris M, Hidalgo G, Baldassano RN, Anokhin AP, Heath AC, Warner B, Reeder J, Kuczynski J, Caporaso JG, Lozupone CA, Lauber C, Clemente JC, Knights D, Knight R, Gordon JI (2012). Human gut microbiome viewed across age and geography. Nature.

[11] Singh P, Teal TK, Marsh TL, Tiedje JM, Mosci R, Jernigan K, Zell A, Newton DW, Salimnia H, Lephart P, Sundin D, Khalife W, Britton RA, Rudrik JT, Manning SD (2015). Intestinal microbial communities associated with acute enteric infections and disease recovery. Microbiome.

[12] Turnbaugh PJ (2009). The effect of diet on the human gut microbiome: a metagenomic analysis in humanized gnotobiotic mice. Sci. Transl. Med..

[13] David LA, Maurice CF, Carmody RN, Gootenberg DB, Button JE, Wolfe BE, Ling AV, Devlin AS, Varma Y, Fischbach MA, Biddinger SB, Dutton RJ, Turnbaugh PJ (2014). Diet rapidly and reproducibly alters the human gut microbiome. Nature.

[14] Dethlefsen L, Relman DA (2011). Incomplete recovery and individualized responses of the human distal gut microbiota to repeated antibiotic perturbation. Proc. Natl. Acad. Sci. U. S. A..

[15] Imhann F, Bonder MJ, Vich Vila A, Fu J, Mujagic Z, Vork L, Tigchelaar EF, Jankipersadsing SA, Cenit MC, Harmsen HJM, Dijkstra G, Franke L, Xavier RJ, Jonkers D, Wijmenga C, Weersma RK, Zhernakova A (2016). Proton pump inhibitors affect the gut microbiome. Gut.

[16] Langelier C, Graves M, Kalantar K, Caldera S, Durrant R, Fisher M, Backman R, Tanner W, DeRisi JL, Leung DT (2019). Microbiome and antimicrobial resistance gene dynamics in international travelers. Emerg. Infect. Dis..

[17] Mehta RS, Abu-Ali GS, Drew DA, Lloyd-Price J, Subramanian A, Lochhead P, Joshi AD, Ivey KL, Khalili H, Brown GT, DuLong C, Song M, Nguyen LH, Mallick H, Rimm EB, Izard J, Huttenhower C, Chan AT (2018). Stability of the human faecal microbiome in a cohort of adult men. Nat. Microbiol..

[18] Faith JJ, Guruge JL, Charbonneau M, Subramanian S, Seedorf H, Goodman AL, Clemente JC, Knight R, Heath AC, Leibel RL, Rosenbaum M, Gordon JI (2013). The long-term stability of the human gut microbiota. Science.

[19] Schloissnig S, Arumugam M, Sunagawa S, Mitreva M, Tap J, Zhu A, Waller A, Mende DR, Kultima JR, Martin J, Kota K, Sunyaev SR, Weinstock GM, Bork P (2013). Genomic variation landscape of the human gut microbiome. Nature.

[20] Franzosa EA, Huang K, Meadow JF, Gevers D, Lemon KP, Bohannan BJM, Huttenhower C (2015). Identifying personal microbiomes using metagenomic codes. Proc. Natl. Acad. Sci. U. S. A..

[21] Andrew T, Hart DJ, Snieder H, de Lange M, Spector TD, MacGregor A (2001). Are twins and singletons comparable? A study of disease-related and lifestyle characteristics in adult women. Twin Res..

[22] Goodrich JK, Waters JL, Poole AC, Sutter JL, Koren O, Blekhman R, Beaumont M, van Treuren W, Knight R, Bell JT, Spector TD, Clark AG, Ley RE (2014). Human genetics shape the gut microbiome. Cell.

[23] Goodrich JK, Davenport ER, Beaumont M, Jackson MA, Knight R, Ober C, Spector TD, Bell JT, Clark AG, Ley RE (2016). Genetic determinants of the gut microbiome in UK twins. Cell Host Microbe.

[24] Xie H (2016). Shotgun metagenomics of 250 adult twins reveals genetic and environmental impacts on the gut microbiome. Cell Syst.

[25] Visconti A, le Roy CI, Rosa F, Rossi N, Martin TC, Mohney RP, Li W, de Rinaldis E, Bell JT, Venter JC, Nelson KE, Spector TD, Falchi M (2019). Interplay between the human gut microbiome and host metabolism. Nat. Commun..

[26] Turnbaugh PJ, Hamady M, Yatsunenko T, Cantarel BL, Duncan A, Ley RE, Sogin ML, Jones WJ, Roe BA, Affourtit JP, Egholm M, Henrissat B, Heath AC, Knight R, Gordon JI (2009). A core gut microbiome in obese and lean twins. Nature.

[27] Bolger AM, Lohse M, Usadel B (2014). Trimmomatic: a flexible trimmer for Illumina sequence data. Bioinformatics.

[28] Li, H. (2013) Aligning sequence reads, clone sequences and assembly contigs with BWA-MEM. *arXiv [q-bio.GN]*

[29] Kim D, Song L, Breitwieser FP, Salzberg SL (2016). Centrifuge: rapid and sensitive classification of metagenomic sequences. Genome Res..

[30] Lander ES, Waterman MS (1988). Genomic mapping by fingerprinting random clones: a mathematical analysis. Genomics.

[31] Truong DT, Tett A, Pasolli E, Huttenhower C, Segata N (2017). Microbial strain-level population structure and genetic diversity from metagenomes. Genome Res..

[32] Albanese D, Donati C (2017). Strain profiling and epidemiology of bacterial species from metagenomic sequencing. Nat. Commun..

[33] Nayfach S, Rodriguez-Mueller B, Garud N, Pollard KS (2016). An integrated metagenomics pipeline for strain profiling reveals novel patterns of bacterial transmission and biogeography. Genome Res..

[34] Li H, Handsaker B, Wysoker A, Fennell T, Ruan J, Homer N, Marth G, Abecasis G, Durbin R, 1000 Genome Project Data Processing Subgroup (2009). The sequence alignment/map format and SAMtools. Bioinformatics.

[35] Koboldt DC, Zhang Q, Larson DE, Shen D, McLellan MD, Lin L, Miller CA, Mardis ER, Ding L, Wilson RK (2012). VarScan 2: somatic mutation and copy number alteration discovery in cancer by exome sequencing. Genome Res..

