# Supplementary material for: Microbial Species that Initially Colonize the Human Gut at Birth or in Early Childhood Can Stay in Human Body for Lifetime
Source: Microb Ecol. 2021 Jan 7;82(4):1074–9. doi: 10.1007/s00248-020-01636-0 (PMC8551085; doi:10.1007/s00248-020-01636-0)
Supplement: Supplementary file 1 — (DOCX 22 kb) [file 248_2020_1636_MOESM1_ESM.docx]

**Table S1**. List of species analyzed in the study

| Species | Representative strain^a^ | Rare indels ^b^ | Rare SNVs ^b^ | Samples ^c^ | MZ pairs ^d^ | DZ pairs ^d^ |
| --- | --- | --- | --- | --- | --- | --- |
| *Akkermansia muciniphila* | *Akkermansia muciniphila* ATCC BAA-835 | 4202 | 167976 | 496 | 33 | 51 |
| *Alistipes putredinis* | *Alistipes putredinis* DSM 17216 | 2669 | 98078 | 487 | 47 | 67 |
| *Alistipes sp. AM16-43* | *Alistipes sp. AM16-43* | 1630 | 92867 | 136 | 0 | 11 |
| *Bacteroides caccae* | *Bacteroides caccae* CL03T12C61 | 3122 | 146962 | 291 | 17 | 25 |
| *Bacteroides dorei* | *Bacteroides dorei* DSM 17855 | 3344 | 140175 | 388 | 31 | 35 |
| *Bacteroides fragilis* | *Bacteroides fragilis* YCH46 | 2729 | 124398 | 144 | 8 | 5 |
| *Bacteroides sp. 4_3_47FAA* | *Bacteroides sp. 4_3_47FAA* | 2772 | 121043 | 211 | 0 | 22 |
| *Bacteroides uniformis* | *Bacteroides uniformis* ATCC 8492 | 5088 | 258615 | 692 | 85 | 116 |
| *Bacteroides vulgatus* | *Bacteroides vulgatus* ATCC 8482 | 3967 | 154113 | 445 | 35 | 0 |
| *Barnesiella intestinihominis* | *Barnesiella intestinihominis* YIT 11860 | 2987 | 109070 | 158 | 9 | 7 |
| *Bifidobacterium adolescentis* | *Bifidobacterium adolescentis* ATCC 15703 | 1840 | 77163 | 234 | 9 | 16 |
| *Bifidobacterium longum* | *Bifidobacterium longum* NCC2705 | 2224 | 84626 | 365 | 13 | 32 |
| *Blautia obeum* | *Blautia obeum* | 4740 | 244203 | 408 | 29 | 0 |
| *Coprococcus comes* | *Coprococcus comes* ATCC 27758 | 3510 | 178464 | 276 | 0 | 20 |
| *Dialister invisus* | *Dialister invisus* DSM 15470 | 3036 | 138556 | 333 | 24 | 30 |
| *Dorea longicatena* | *Dorea longicatena* DSM 13814 | 2520 | 135929 | 405 | 24 | 30 |
| *Escherichia coli* | *Escherichia coli* O104:H4 str. 2011C-3493 | 4197 | 325187 | 201 | 7 | 6 |
| *Eubacterium hallii* | *Eubacterium hallii* DSM 3353 | 2897 | 124141 | 333 | 20 | 0 |
| *Eubacterium rectale* | *Eubacterium rectale* ATCC 33656 | 4818 | 252325 | 375 | 22 | 0 |
| *Eubacterium siraeum* | *Eubacterium siraeum* DSM 15702 | 3944 | 185371 | 198 | 8 | 14 |
| *Gemmiger formicilis* | *Gemmiger formicilis* | 5974 | 351462 | 212 | 0 | 10 |
| *Methanobrevibacter smithii* | *Methanobrevibacter smithii* ATCC 35061 | 3387 | 119174 | 237 | 9 | 13 |
| *Parabacteroides merdae* | *Parabacteroides merdae* ATCC 43184 | 3764 | 160700 | 255 | 11 | 21 |
| *Phascolarctobacterium faecium* | *Phascolarctobacterium faecium* DSM 14760 | 1595 | 95989 | 199 | 10 | 10 |
| *Ruminococcaceae bacterium TF06-43* | *Ruminococcaceae bacterium TF06-43* | 4643 | 291271 | 229 | 11 | 10 |
| *Streptococcus thermophilus* | *Streptococcus thermophilus* JIM 8232 | 2216 | 103649 | 181 | 7 | 6 |
| *Subdoligranulum sp. APC924/74* | *Subdoligranulum sp. APC924/74* | 8008 | 380026 | 508 | 44 | 56 |
| *^a^ The genome of this strain was used in reads mapping to derive SNVs and indels*  *^b^ Rare SNVs or indels are those only exist in less than 20% of samples that contain this species at high coverage (see methods)*  *^c^ Number of samples that contain this species at high coverage*  *^d^ Number of twin pairs that contain this species at high coverage* | | | | | | |
